# Supplementary material for: Entrenchment and contingency in neutral protein evolution with epistasis
Source: bioRxiv. 2025 Jan 14:2025.01.09.632266. Preprint. [Version 1] doi: 10.1101/2025.01.09.632266 (PMC11761135; doi:10.1101/2025.01.09.632266)
Supplement: Supplement 1 [file media-1.pdf]

Title: Entrenchment and contingency in neutral protein evolution with epistasis

Authors: Lisa Schmelkin[1,2], Vincenzo Carnevale[1,2,3], Allan Haldane[3,4,5], Jeffrey P. Townsend[6], Sarah Chung[1,2], Ronald M. Levy[3,4,5], and Sudhir Kumar[*,1,2]

Supplementary Materials

Methods

Constructing ortho-domain alignments

We retrieved a total of 19 different protein domain family alignments analyzed in de la Paz et al. (*5*) and Figliuzzi et al. (*25*). For each family, we identified vertebrate orthologs by first searching for member sequences within the human proteome. We constructed a database of human proteins by selecting the human sequence from 19,077 UCSC 100-vertebrate alignments (*15*) and running individual BLAST searches for each of the domain families. Since we have a human to 100-vertebrate mapping within the alignments, it suffices to identify domains only within the human protein-coding sequences and then retrieve vertebrate orthologs of the same positions. We only consider hits with 100% sequence coverage and do not retain domains with insertions and deletions because the number of positions must match that of the family alignment in order to accurately calculate φ. Therefore, each ortho-domain alignment will contain up to 100 vertebrate species, but sequences may be removed when calculating the neutral range in energy (φ). After locating the positions of the domain in the vertebrate alignment, we removed all sites that were not part of the identified domain and sequences with missing data. We were able to construct over 300 ortho-domain alignments spanning a total of six different protein domain families (**Table S3**).

For domain families analyzed by the SEEC framework (*5*), the MSA and associated pairwise coupling (*e*) and local fields (*h*) matrices were extracted from the "Parameters_orig-x.mat" files made available by the authors, so no further PH model parameter inference was necessary (*https://doi.org/10.5061/dryad.2ngf1vhj8*). For the additional domain families analyzed in Figliuzzi et al. (*25*), we ran the bmDCA algorithm (*https://github.com/matteofigliuzzi/bmDCA*) on the provided MSAs of domain homologs to obtain *e* and *h* matrices.

Potts Hamiltonian (PH) model construction

Direct Coupling Analysis (DCA; *4*) is an umbrella term for the analysis of direct pairwise epistasis within a large MSA of homologous domains in order to produce a joint probability model, of which Potts Hamiltonian (PH) models are one variant. Some details of our simulations may depend on details of the PH models used in this analysis. Alternative PH inference algorithms make different approximations and yield somewhat different PH parameterizations when fit to the same MSA data. For instance, it was found that SEEC simulations differed when using PH models fit using mfDCA instead of bmDCA, and in some SEEC models a "selection temperature" can be tuned to adjust the simulation statistics (*24*). Many PH inference methods exist, but methods such as Mi3-GPU and bmDCA involving MCMC sampling of the model posterior distribution Boltzmann machine learning DCA (*25, 35, 36*) have been validated to capture the residue variability of the domain family with high precision. During inference of such PH models, clusters of orthologs with high sequence identity are downweighted, typically to have an effective count

of 1 each. For PF00001 this left 6,593 effective sequences after weighting at an 80% sequence identity threshold, roughly representing an effective MSA of this many paralogous sequences.

The two components of the inferred PH model are a matrix of site-specific amino acid preferences (local fields; *h*) and a matrix of pairwise couplings (*e*) containing preferences for every pair of sites. From these components, Potts Hamiltonian Energy (PHE; φ) can be calculated for any sequence native to the protein domain family, as

$$P(\sigma_i = \alpha) = \frac{1}{Z} \exp \left\{ h_i(\alpha) + \sum_{j \neq i} e_{ij}(\alpha, \sigma_j) \right\} \qquad (1)$$

In **Eq. 1**, *e* and *h* refer to the matrices as above, which are evaluated for each amino acid σ at site i (σ_i) and all possible pairs of sites (σ_i, σ_j) for a sequence with N total positions.

Simulating ortho-domain evolution

To simulate ortho-domain evolution, we begin by following the procedure of the SEEC model (*5*). At each evolutionary step, we randomly sample a site along the protein by a uniform distribution where each site has an equal probability of being selected. We then sample an amino acid (including the probability of a synonymous change) from a conditional probability distribution (**Eq 2**), which describes the likelihood of finding each amino acid at the sampled position where the rest of the sequence is constant. This Boltzmann probability distribution treats each domain as a multivariate random variable and describes the probability of the sequence in relation to its family members, where *Z* is a normalizing constant.

$$\varphi = -\sum_{i=1}^{N-1} \sum_{j=i+1}^{N} e_{ij}(\sigma_i, \sigma_j) - \sum_{i=1}^{N} h_i(\sigma_i) \qquad (2)$$

In other words, each possible residue (or an alignment gap character) is inserted into the sequence at the site being considered, and a distribution is constructed to find the instantaneous probabilities of substitution. While more probable sequences are favored, by the nature of the sampling procedure, there is still a chance of sampling less likely residues.

After a substitution is sampled, we calculate φ of the evolved domain. We assess if the substitution is predicted to be *neutral* as determined by the range in φ of sequences within the empirical ortho-domain alignment (see **Table S1** for PF00001 ortho-domain ranges). If φ falls outside the neutral range, we resample a site and residue by the above procedure until a permissible substitution is found. Although resampling occurs, when we track an evolutionary trajectory, only the permitted substitution and domain sequence are recorded.

Justification for imposing a strict min-max boundary in φ (neutral zone)

When we constructed alignments of ortho-domains in vertebrate species (UCSC 100-vertebrate alignments, see above), sequences had to be removed due to indels and missing data. Therefore, in many cases, the range in φ could not be calculated from all 100 sequences. Even when all data were available, the number of empirical sequences is limited to fully capture the statistical energies expected among domain orthologs. Therefore, for each of the 78 PF00001 ortho-domains, we calculated a normalized distribution of energies by finding the mean and variance of observations. We then found the percentage of the normalized distribution that was contained by the φ_{min} and

φ_{max} that were calculated from the empirical orthologs. On average, 92.1% of the distribution was contained (range 78.0–99.6%).

Star phylogeny simulation procedure and calculating R

We simulate star phylogenies following Fitch & Margoliash (*37*) and Kimura & Ohta (*26*) to avoid phylogenetic correlations among species. We begin with a sequence native to an ortho-domain alignment and simulate 100 lineages (replicates) emanating from the common ancestor. Lineages are evolved independently with all PH model parameters kept constant and the range in φ restricted to that of the empirical orthologs.

Star phylogenies have been classically used to test the molecular clock hypothesis by finding the number of substitutions that have accumulated in each species and computing *R* as the ratio of the variance to the mean. This calculation makes the underlying assumptions that the rate of all mutations and the selection intensity remain constant across species and over time. In N×E simulations, selection intensity remains constant because it is induced by the PH model and restriction in φ, both of which are constant among lineages in the tree. But, we must impose a constant mutation rate. If the mutation rate is constant among species, we expect that the *total number* of mutations in each lineage will be Poisson-distributed. Since each evolutionary step in N×E simulations either results in a synonymous or nonsynonymous substitution, it is sufficient to sample the number of evolutionary steps in each branch of the tree from a Poisson distribution with the same expectation. For each simulated tree, the mean number of evolutionary steps is chosen based on the purifying selection rate in the given ortho-domain and the desired evolutionary distance (e.g., $d = 0.25$ or 0.5 substitutions per site).

We test these conditions in a control experiment without pairwise couplings (independent-site evolution) and a random evolution model in which all residues are equally likely to appear (uniform evolution). As expected, R=1 on average with minimal variance regardless of evolutionary distance. Therefore, any deviation from R=1 for N×E simulations will be due to pairwise epistasis.

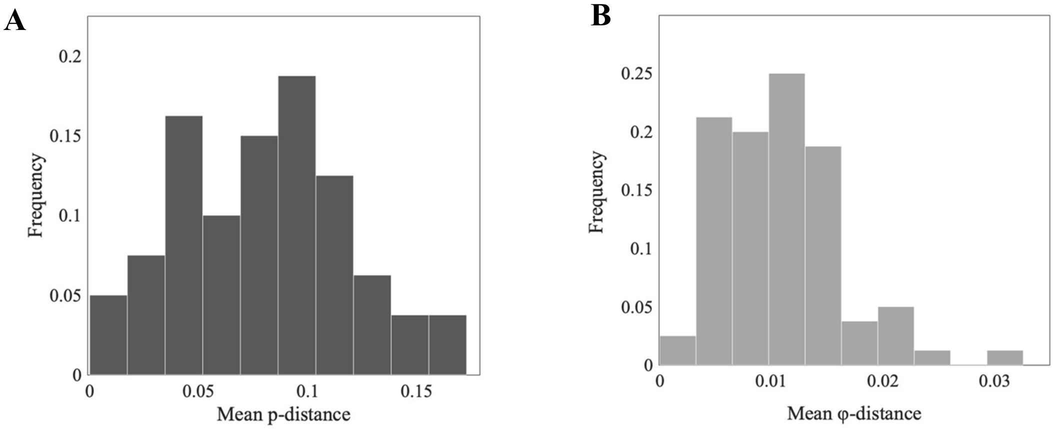

Figure S1. (**A**) Average *p*-distance for pairs of orthologs for each of the 78 variable PF00001 ortho-domain alignments (**B**) Average φ-distance for pairs of orthologs for each of the 78 variable PF00001 ortho-domain alignments

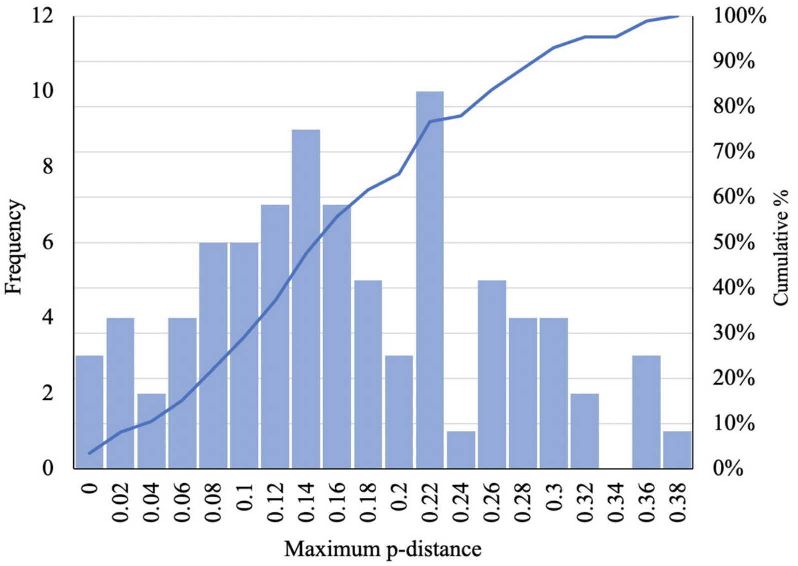

Figure S2. Maximum *p*-distance within PF00001 ortho-domains. For each of the 78 variable protein-coding genes with at least 1 copy of PF00001, we found the maximum *p*-distance among vertebrate orthologs. *p*-distance (Hamming distance) is calculated as the proportion of sites at which the two sequences are different in comparison to amino acid residues and alignment gaps.

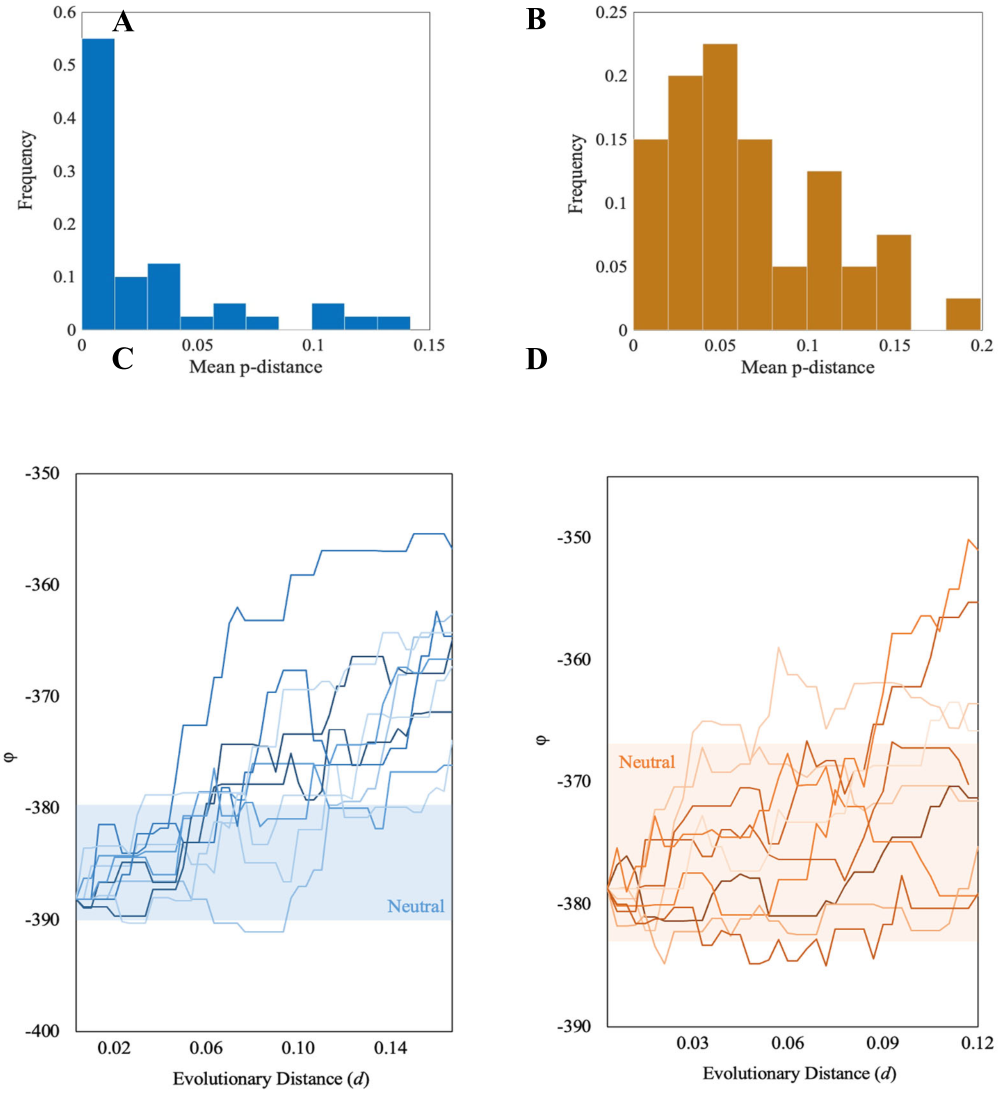

Figure S3. Average *p*-distance among orthologs for PF0004 and PF00005 and φ trajectories. (**A–B**) Average *p*-distance within ortho-domains (up to 100 vertebrate species) in each family (PF00004 and PF00005 respectively). (**C–D**) Trajectories of φ for single lineages evolved to *p*-distance = 0.1

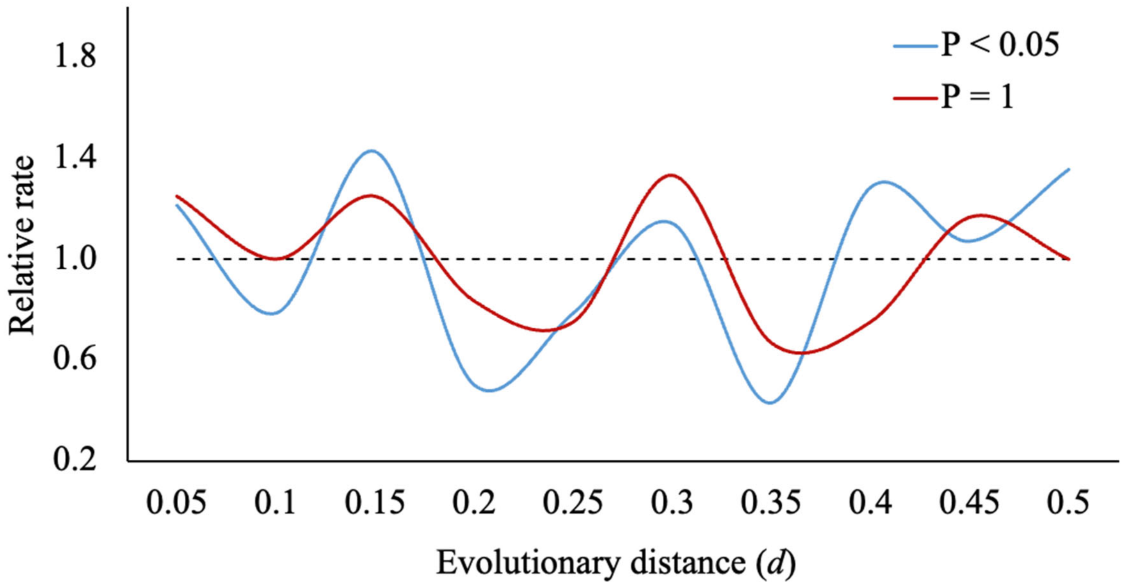

Figure S4. Rate oscillation for a lineage that rejects the null hypothesis of no contingency. Example of 2 lineages within the PTGER4 star tree: one that rejects the null hypothesis of no contingency ($P < 0.05$) and one that does not ($P = 1$). Despite evolutionary rates being non-random, there is not a propensity for slow-evolving lineages to remain slow-evolving or vice versa. Instead, rates oscillate predictably from slow- to fast-evolving in the tree.

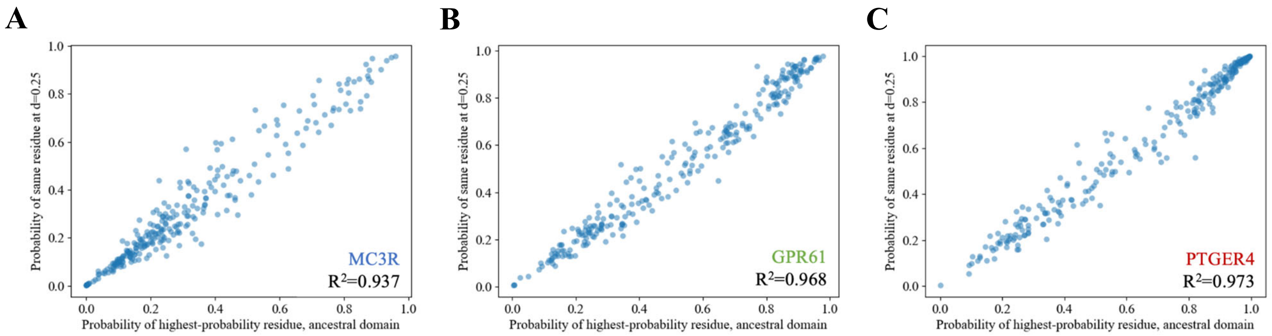

Figure S5. Amino-acid propensities for ancestor and descendent at d = 0.25 (MC3R, GPR61 and PTGER4 proteins). (**A**) Probability of highest-probability residue at ancestor versus probability of same residue at descendant evolved to *d* = 0.25 substitutions per site (PF00001 domain found in the MC3R protein; R^2 = 0.937). (**B**) Probability of highest-probability residue at ancestor versus probability of same residue at descendant evolved to d = 0.25 substitutions per site (PF00001 domain found in the GPR61 protein; R^2 = 0.968). (**C**) Probability of highest-probability residue at ancestor versus probability of same residue at descendant evolved to d = 0.25 substitutions per site (PF00001 domain found in the PTGER4 protein; R^2 = 0.973).

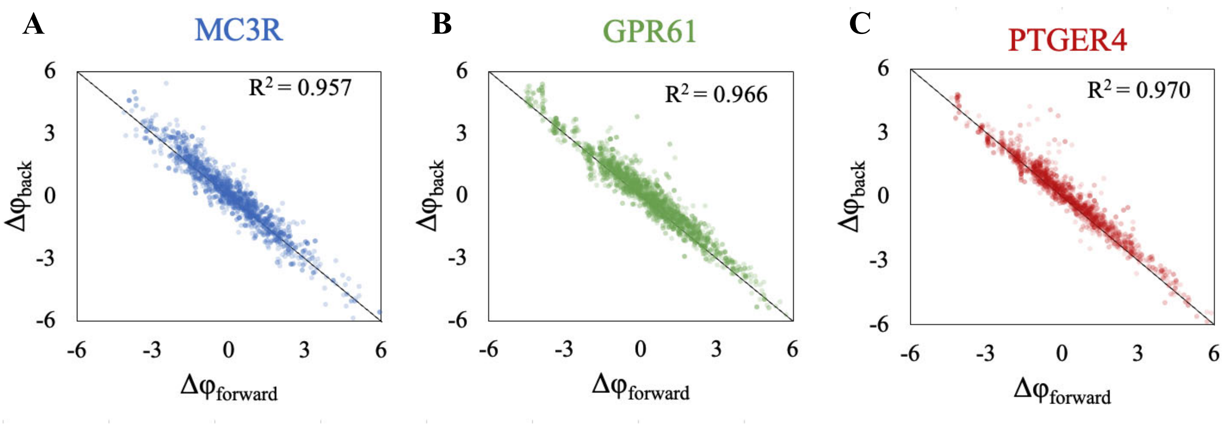

Figure S6. Stokes shift at d = 0.5 substitutions per site. (**A–C**) Stokes shift plots of $\Delta\varphi_{forward}$ and $\Delta\varphi_{back}$ following the methods of de la Paz et al. (*5*). Each plot includes every site which differed from ancestor to descendent (d = 0.5) across 30 different replicates. Calculations were done for PF00001 ortho-domains with low |φ| (MC3R), median φ (GPR61) and high |φ| (PTGER4), respectively.
%

Table S1. Summary of statistical energies φ of PF00001 ortho-domains in 78 proteins. Provided is the NMID of the alignment as retrieved from the UCSC 100-vertebrate alignments (*15*), gene name, and summary of φ values as calculated from the PH model provided by de la Paz et al. (*5*).

Protein	NMID	Min φ	Max φ	Mean φ	φ Range
**PTGER4**	NM_000958	-704.822158	-693.732259	-702.094	11.089899
**TRHR**	NM_003301	-720.473822	-681.857128	-692.069	38.616694
**ADRB2**	NM_000024	-688.696522	-672.099206	-683.130	16.597316
**NMUR1**	NM_006056	-692.52532	-668.995698	-680.908	23.529622
**AVPR1A**	NM_000706	-697.726517	-648.722949	-675.002	49.003568
**ADORA2A**	NM_000675	-691.759314	-651.351909	-674.511	40.407405
**OXTR**	NM_000916	-674.863732	-655.212697	-666.436	19.651035
**ADRB1**	NM_000684	-663.765905	-663.765905	-663.766	0
**DRD5**	NM_000798	-656.084801	-648.21986	-652.438	7.864941
**AVPR1B**	NM_000707	-664.938444	-631.635449	-650.842	33.302995
**DRD1**	NM_000794	-647.446488	-630.19813	-638.817	17.248358
**TAAR1**	NM_138327	-662.805856	-604.864591	-637.869	57.941265
**OPN4**	NM_033282	-640.418662	-600.199931	-631.237	40.218731
**ADRA1B**	NM_000679	-634.997964	-619.338979	-630.591	15.658985
**NPFFR2**	NM_004885	-640.013065	-592.806112	-628.540	47.206953
**HTR2C**	NM_000868	-633.636532	-604.714545	-628.298	28.921987
**ADRA1A**	NM_000680	-625.855902	-601.952131	-616.990	23.903771
**HTR1F**	NM_001322209	-624.6765	-597.37581	-615.701	27.30069
**NPFFR1**	NM_022146	-619.441135	-602.015996	-614.087	17.425139
**ADRA1D**	NM_000678	-616.486901	-605.696747	-613.438	10.790154
**GPR26**	NM_153442	-614.202656	-598.716322	-609.258	15.486334
**HTR2A**	NM_000621	-612.502925	-588.297558	-607.994	24.205367
**ADRB3**	NM_000025	-620.565076	-589.878742	-607.907	30.686334
**HTR1D**	NM_000864	-616.249939	-590.971167	-606.551	25.278772
**HTR1B**	NM_000863	-609.62804	-594.598203	-604.647	15.029837
**AVPR2**	NM_000054	-614.947365	-585.17309	-604.078	29.774275
**HTR1E**	NM_000865	-608.040499	-597.286776	-602.228	10.753723
**ADORA1**	NM_000674	-610.187239	-557.330653	-601.037	52.856586
**GRPR**	NM_005314	-601.819953	-572.074781	-592.932	29.745172
**PTGIR**	NM_000960	-584.612102	-576.479235	-581.304	8.132867
**NTSR1**	NM_002531	-594.282586	-552.947237	-576.998	41.335349
**NMBR**	NM_002511	-585.986498	-558.498099	-575.486	27.488399
**GPR39**	NM_001508	-585.256359	-560.164315	-574.270	25.092044
**HTR2B**	NM_000867	-597.06396	-556.233717	-572.160	40.830243
**CCKAR**	NM_000730	-581.542831	-565.118093	-571.481	16.424738
**HTR7**	NM_019859	-576.719138	-541.186178	-571.088	35.53296

**GPR61**	NM_001393907	-584.134718	-562.129484	-571.072	22.005234
**CCKBR**	NM_176875	-575.492677	-562.978823	-567.888	12.513854
**HTR4**	NM_000870	-564.726375	-552.406872	-556.948	12.319503
**BRS3**	NM_001727	-573.419086	-536.063953	-556.695	37.355133
**CNR2**	NM_001841	-569.277584	-520.370321	-555.216	48.907263
**HCRTR2**	NM_001384272	-550.753268	-513.257146	-543.166	37.496122
**GNRHR**	NM_000406	-553.39427	-527.882972	-539.351	25.511298
**HTR5A**	NM_024012	-546.59413	-515.733056	-535.287	30.861074
**PTGER2**	NM_000956	-554.553623	-478.730826	-535.117	75.822797
**HTR6**	NM_000871	-540.847969	-521.088287	-534.746	19.759682
**KISS1R**	NM_032551	-545.062762	-524.354565	-534.230	20.708197
**S1PR1**	NM_001400	-541.052965	-516.56726	-531.744	24.485705
**PRLHR**	NM_004248	-537.401726	-496.769486	-525.915	40.63224
**HCRTR1**	NM_001525	-530.763096	-512.841604	-525.529	17.921492
**FFAR4**	NM_001195755	-536.880945	-495.35399	-522.654	41.526955
**CNR1**	NM_016083	-521.955649	-501.757257	-517.554	20.198392
**GPR78**	NM_080819	-521.441843	-507.128897	-514.702	14.312946
**S1PR5**	NM_030760	-522.457616	-499.238256	-514.519	23.21936
**GPR22**	NM_005295	-511.392154	-511.392154	-511.392	0
**GPR119**	NM_178471	-522.826139	-487.63306	-509.890	35.193079
**NPSR1**	NM_207172	-515.718437	-476.513518	-505.977	39.204919
**GPR161**	NM_001375883	-509.407089	-490.766993	-503.160	18.640096
**PTGER3**	NM_198719	-509.799806	-491.021836	-501.179	18.77797
**GPR45**	NM_007227	-508.291431	-470.964631	-498.712	37.3268
**QRFPR**	NM_198179	-506.526335	-479.68712	-497.681	26.839215
**GPR85**	NM_001146267	-501.799348	-485.280019	-491.377	16.519329
**PTGER1**	NM_000955	-499.375806	-475.335872	-489.641	24.039934
**GPR173**	NM_018969	-488.977755	-473.330441	-486.032	15.647314
**NTSR2**	NM_012344	-493.140817	-447.261111	-480.036	45.879706
**MCHR2**	NM_001040179	-485.520459	-455.722087	-479.979	29.798372
**GPR63**	NM_030784	-485.100715	-463.161765	-478.738	21.93895
**GPR21**	NM_005294	-490.463064	-467.361737	-478.242	23.101327
**GPR19**	NM_006143	-482.315808	-457.000409	-474.319	25.315399
**GHSR**	NM_198407	-483.44077	-459.034155	-472.400	24.406615
**GPR52**	NM_005684	-474.629632	-455.109244	-468.295	19.520388
**RXFP3**	NM_016568	-471.716636	-449.127921	-463.732	22.588715
**GPR27**	NM_018971	-456.525267	-448.917292	-454.160	7.607975
**GPR135**	NM_022571	-453.779091	-433.135369	-444.387	20.643722
**LPAR3**	NM_012152	-432.815129	-410.058791	-425.050	22.756338
**GPR88**	NM_022049	-425.258179	-415.368198	-423.002	9.889981
**GPR176**	NM_007223	-426.513221	-394.05316	-420.666	32.460061

**MC3R**	NM_019888	-415.515959	-400.307407	-408.277	15.208552
**GPR62**	NM_080865	-408.897214	-396.495607	-404.366	12.401607
**GPR162**	NM_019858	-382.930692	-378.225139	-380.697	4.705553

Table S2. Mean *R* values for star trees evolved to d = 0.5 substitutions per site, emanating from each of the 78 variable PF00001 ortho-domains.

Protein	Mean *R*
ADRB2	1.19950037
ADRB3	1.07765429
AVPR2	1.06494963
GNRHR	1.06714507
HTR2A	1.23772661
ADORA1	1.0243695
ADORA2A	1.19075793
ADRA1D	1.27324121
ADRA1B	1.34544148
ADRA1A	1.29503672
AVPR1A	1.16103349
AVPR1B	1.10831582
CCKAR	1.21343401
DRD1	1.40240792
DRD5	1.45929035
HTR1B	1.11819071
HTR1D	1.18984456
HTR1E	1.18663543
HTR2B	1.26832556
HTR2C	1.21384758
HTR4	1.03987682
HTR6	1.10465527
OXTR	1.22072697
PTGER1	1.11081073
PTGER2	1.03056926
PTGER4	1.2487132
PTGIR	1.17583453
MCHR2	1.22142575

GPR85	1.22961445
FFAR4	1.14155954
HTR1F	1.07210193
GPR161	1.01508891
HCRTR2	1.12556558
GPR61	1.02783205
S1PR1	1.12874618
GPR39	1.3249735
HCRTR1	1.29962317
BRS3	1.2460244
CNR2	1.20032996
NMBR	1.12040204
NTSR1	1.27203834
TRHR	1.31554914
PRLHR	1.11631746
NPFFR2	1.13648622
GPR21	1.32916204
GRPR	1.22726854
GPR52	1.04253974
NMUR1	1.19053517
GPR19	1.08153177
GPR176	1.11424318
GPR45	1.18534026
LPAR3	1.42515233
NTSR2	1.21318888
CNR1	1.27418078
RXFP3	1.21823715
GPR173	1.07472834
GPR27	1.08249569
GPR162	1.22547281
HTR7	1.04220032

MC3R	1.08756273
GPR88	1.17363904
NPFFR1	1.17754722
GPR135	1.33310312
HTR5A	1.16753649
S1PR5	1.08499855
GPR63	1.03335117
KISS1R	0.98193454
OPN4	1.11271493
GPR78	1.14242065
GPR62	1.08749376
TAAR1	1.26308816
GPR26	1.23921249
CCKBR	1.20207787
GPR119	1.1255193
QRFPR	1.03473346
GHSR	1.08414148
PTGER3	1.25125275
NPSR1	1.10083037

Table S3. Summary of ortho-domains constructed for 6 different protein domain families, mean energy (φ), and maximum φ-distance among sequences.

Pfam ID	Pfam Short Name	Protein	NMID	Mean φ	Max φ-dist
PF00001	7tm_1	ADRB2	NM_000024	-683.13	0.02
PF00001	7tm_1	ADRB3	NM_000025	-607.91	0.05
PF00001	7tm_1	AVPR2	NM_000054	-604.08	0.05
PF00001	7tm_1	GNRHR	NM_000406	-539.35	0.05
PF00001	7tm_1	HTR2A	NM_000621	-607.99	0.04
PF00001	7tm_1	ADORA1	NM_000674	-601.04	0.09
PF00001	7tm_1	ADORA2A	NM_000675	-674.51	0.06
PF00001	7tm_1	ADRA1D	NM_000678	-613.44	0.02
PF00001	7tm_1	ADRA1B	NM_000679	-630.59	0.02
PF00001	7tm_1	ADRA1A	NM_000680	-616.99	0.04
PF00001	7tm_1	ADRB1	NM_000684	-663.77	0
PF00001	7tm_1	AVPR1A	NM_000706	-675	0.07
PF00001	7tm_1	AVPR1B	NM_000707	-650.84	0.05
PF00001	7tm_1	CCKAR	NM_000730	-571.48	0.03
PF00001	7tm_1	DRD1	NM_000794	-638.82	0.03
PF00001	7tm_1	DRD5	NM_000798	-652.44	0.01
PF00001	7tm_1	HTR1B	NM_000863	-604.65	0.02
PF00001	7tm_1	HTR1D	NM_000864	-606.55	0.04
PF00001	7tm_1	HTR1E	NM_000865	-602.23	0.02
PF00001	7tm_1	HTR2B	NM_000867	-572.16	0.07
PF00001	7tm_1	HTR2C	NM_000868	-628.3	0.05
PF00001	7tm_1	HTR4	NM_000870	-556.95	0.02
PF00001	7tm_1	HTR6	NM_000871	-534.75	0.04
PF00001	7tm_1	OXTR	NM_000916	-666.44	0.03
PF00001	7tm_1	PTGER1	NM_000955	-489.64	0.05
PF00001	7tm_1	PTGER2	NM_000956	-535.12	0.14
PF00001	7tm_1	PTGER4	NM_000958	-702.09	0.02
PF00001	7tm_1	PTGIR	NM_000960	-581.3	0.01
PF00001	7tm_1	MCHR2	NM_001040179	-479.98	0.06
PF00001	7tm_1	GPR85	NM_001146267	-491.38	0.03
PF00001	7tm_1	FFAR4	NM_001195755	-522.65	0.08
PF00001	7tm_1	HTR1F	NM_001322209	-615.7	0.04
PF00001	7tm_1	GPR161	NM_001375883	-503.16	0.04
PF00001	7tm_1	HCRTR2	NM_001384272	-543.17	0.07
PF00001	7tm_1	GPR61	NM_001393907	-571.07	0.04
PF00001	7tm_1	S1PR1	NM_001400	-531.74	0.05
PF00001	7tm_1	GPR39	NM_001508	-574.27	0.04
PF00001	7tm_1	HCRTR1	NM_001525	-525.53	0.03
PF00001	7tm_1	BRS3	NM_001727	-556.7	0.07
PF00001	7tm_1	CNR2	NM_001841	-555.22	0.09

PF00001	7tm_1	NMBR	NM_002511	-575.49	0.05
PF00001	7tm_1	NTSR1	NM_002531	-577	0.07
PF00001	7tm_1	TRHR	NM_003301	-692.07	0.06
PF00001	7tm_1	PRLHR	NM_004248	-525.91	0.08
PF00001	7tm_1	NPFFR2	NM_004885	-628.54	0.08
PF00001	7tm_1	GPR21	NM_005294	-478.24	0.05
PF00001	7tm_1	GPR22	NM_005295	-511.39	0
PF00001	7tm_1	GRPR	NM_005314	-592.93	0.05
PF00001	7tm_1	GPR52	NM_005684	-468.29	0.04
PF00001	7tm_1	NMUR1	NM_006056	-680.91	0.03
PF00001	7tm_1	GPR19	NM_006143	-474.32	0.05
PF00001	7tm_1	GPR176	NM_007223	-420.67	0.08
PF00001	7tm_1	GPR45	NM_007227	-498.71	0.07
PF00001	7tm_1	LPAR3	NM_012152	-425.05	0.05
PF00001	7tm_1	NTSR2	NM_012344	-480.04	0.1
PF00001	7tm_1	CNR1	NM_016083	-517.55	0.04
PF00001	7tm_1	RXFP3	NM_016568	-463.73	0.05
PF00001	7tm_1	GPR173	NM_018969	-486.03	0.03
PF00001	7tm_1	GPR27	NM_018971	-454.16	0.02
PF00001	7tm_1	GPR162	NM_019858	-380.7	0.01
PF00001	7tm_1	HTR7	NM_019859	-571.09	0.06
PF00001	7tm_1	MC3R	NM_019888	-408.28	0.04
PF00001	7tm_1	GPR88	NM_022049	-423	0.02
PF00001	7tm_1	NPFFR1	NM_022146	-614.09	0.03
PF00001	7tm_1	GPR135	NM_022571	-444.39	0.05
PF00001	7tm_1	HTR5A	NM_024012	-535.29	0.06
PF00001	7tm_1	S1PR5	NM_030760	-514.52	0.05
PF00001	7tm_1	GPR63	NM_030784	-478.74	0.05
PF00001	7tm_1	KISS1R	NM_032551	-534.23	0.04
PF00001	7tm_1	OPN4	NM_033282	-631.24	0.06
PF00001	7tm_1	GPR78	NM_080819	-514.7	0.03
PF00001	7tm_1	GPR62	NM_080865	-404.37	0.03
PF00001	7tm_1	TAAR1	NM_138327	-637.87	0.09
PF00001	7tm_1	GPR26	NM_153442	-609.26	0.03
PF00001	7tm_1	CCKBR	NM_176875	-567.89	0.02
PF00001	7tm_1	GPR119	NM_178471	-509.89	0.07
PF00001	7tm_1	QRFPR	NM_198179	-497.68	0.05
PF00001	7tm_1	GHSR	NM_198407	-472.4	0.05
PF00001	7tm_1	PTGER3	NM_198719	-501.18	0.04
PF00001	7tm_1	NPSR1	NM_207172	-505.98	0.08
PF00004	*AAA*	PEX6	NM_000287	-378.65	0.1
PF00004	*AAA*	PEX1	NM_000466	-371.69	0.06
PF00004	*AAA*	ATAD3C	NM_001039211	-350.83	0.24

PF00004	*AAA*	BCS1L	NM_001079866	-356.45	0.04
PF00004	*AAA*	ATAD3A	NM_001170535	-353.29	0.07
PF00004	*AAA*	FIGNL1	NM_001287492	-386.54	0.05
PF00004	*AAA*	IQCA1L	NM_001304419	-335.75	0.13
PF00004	*AAA*	FIGNL2	NM_001384995	-260.72	0.03
PF00004	*AAA*	KATNAL2	NM_001387690	-357.79	0.05
PF00004	*AAA*	NVL	NM_002533	-367.42	0.04
PF00004	*AAA*	PSMC1	NM_002802	-363.68	0.02
PF00004	*AAA*	PSMC2	NM_002803	-340.02	0
PF00004	*AAA*	PSMC3	NM_002804	-205.18	0.01
PF00004	*AAA*	PSMC5	NM_002805	-372.87	0
PF00004	*AAA*	PSMC6	NM_002806	-324.6	0.02
PF00004	*AAA*	RFC1	NM_002913	-348.11	0.04
PF00004	*AAA*	RFC4	NM_002916	-368	0.04
PF00004	*AAA*	SPG7	NM_003119	-344.87	0.03
PF00004	*AAA*	ORC1	NM_004153	-342.59	0.05
PF00004	*AAA*	TRIP13	NM_004237	-391.08	0.03
PF00004	*AAA*	LONP1	NM_004793	-378.74	0.04
PF00004	*AAA*	VPS4B	NM_004869	-154.63	0.07
PF00004	*AAA*	NSF	NM_006178	-382.1	0.05
PF00004	*AAA*	PSMC4	NM_006503	-356.68	0
PF00004	*AAA*	AFG3L2	NM_006796	-373.38	0.03
PF00004	*AAA*	KATNA1	NM_007044	-375.08	0.03
PF00004	*AAA*	VCP	NM_007126	-314.42	0.01
PF00004	*AAA*	RFC5	NM_007370	-189.29	0.03
PF00004	*AAA*	VPS4A	NM_013245	-379.44	0.04
PF00004	*AAA*	ATAD2	NM_014109	-334	0.04
PF00004	*AAA*	YME1L1	NM_014263	-358.3	0.15
PF00004	*AAA*	SPAST	NM_014946	-388.69	0.03
PF00004	*AAA*	ATAD2B	NM_017552	-329.78	0.03
PF00004	*AAA*	FIGN	NM_018086	-281.18	0.05
PF00004	*AAA*	WRNIP1	NM_020135	-374.96	0.07
PF00004	*AAA*	SPATA5L1	NM_024063	-320.95	0.06
PF00004	*AAA*	IQCA1	NM_024726	-184.92	0.12
PF00004	*AAA*	LONP2	NM_031490	-384.08	0.04
PF00004	*AAA*	ATAD3B	NM_031921	-350.56	0.23
PF00004	*AAA*	KATNAL1	NM_032116	-376.25	0.03
PF00004	*AAA*	SPATA5	NM_145207	-359.87	0.05
PF00004	*AAA*	RFC2	NM_181471	-200.08	0.04
PF00005	ABC_tran	ABCD1	NM_000033	-376.49	0.08
PF00005	ABC_tran	ABCA4	NM_000350	-375.79	0.14
PF00005	ABC_tran	ABCC8	NM_000352	-373.05	0.06
PF00005	ABC_tran	ABCC2	NM_000392	-372.84	0.07

PF00005	ABC_tran	ABCB4	NM_000443	-370.59	0.05
PF00005	ABC_tran	CFTR	NM_000492	-366.43	0.1
PF00005	ABC_tran	TAP1	NM_000593	-365.76	0.05
PF00005	ABC_tran	ABCF1	NM_001025091	-365.04	0.08
PF00005	ABC_tran	ABCA3	NM_001089	-359.8	0.14
PF00005	ABC_tran	ABCB5	NM_001163941	-356.37	0.09
PF00005	ABC_tran	ABCC6	NM_001171	-355.8	0.07
PF00005	ABC_tran	ABCC10	NM_001198934	-354.47	0.09
PF00005	ABC_tran	ABCB7	NM_001271696	-353.42	0.05
PF00005	ABC_tran	ABCA8	NM_001288985	-351.49	0.04
PF00005	ABC_tran	TAP2	NM_001290043	-349.37	0.07
PF00005	ABC_tran	ABCB1	NM_001348946	-339.56	0.06
PF00005	ABC_tran	ABCC11	NM_001370497	-339.23	0.09
PF00005	ABC_tran	ABCA10	NM_001377321	-337.89	0.04
PF00005	ABC_tran	ABCC12	NM_001393797	-337.65	0.03
PF00005	ABC_tran	ABCA2	NM_001606	-336.19	0.09
PF00005	ABC_tran	ABCD3	NM_002858	-333.86	0.08
PF00005	ABC_tran	ABCE1	NM_002940	-326.09	0.12
PF00005	ABC_tran	ABCB11	NM_003742	-325.67	0.06
PF00005	ABC_tran	ABCC3	NM_003786	-324.07	0.09
PF00005	ABC_tran	ABCG2	NM_004827	-321.85	0.1
PF00005	ABC_tran	ABCC1	NM_004996	-320.55	0.03
PF00005	ABC_tran	ABCD4	NM_005050	-318.54	0.08
PF00005	ABC_tran	ABCD2	NM_005164	-316.77	0.03
PF00005	ABC_tran	ABCA1	NM_005502	-305.5	0.03
PF00005	ABC_tran	ABCC5	NM_005688	-302.97	0.03
PF00005	ABC_tran	ABCB6	NM_005689	-300.44	0.06
PF00005	ABC_tran	ABCC4	NM_005845	-293.81	0.1
PF00005	ABC_tran	ABCB8	NM_007188	-293.61	0.08
PF00005	ABC_tran	ABCF2	NM_007189	-292.94	0.1
PF00005	ABC_tran	ABCB10	NM_012089	-292.84	0.13
PF00005	ABC_tran	ABCG1	NM_016818	-291.09	0.13
PF00005	ABC_tran	ABCF3	NM_018358	-289.37	0.11
PF00005	ABC_tran	ABCA7	NM_019112	-289.06	0.11
PF00005	ABC_tran	ABCB9	NM_019625	-285.59	0.07
PF00005	ABC_tran	ABCC9	NM_020297	-284.79	0.06
PF00005	ABC_tran	ABCG4	NM_022169	-275.56	0.05
PF00005	ABC_tran	ABCG5	NM_022436	-271.15	0.04
PF00005	ABC_tran	ABCG8	NM_022437	-241.23	0.05
PF00005	ABC_tran	ABCA9	NM_080283	-158.85	0.05
PF00005	ABC_tran	ABCA5	NM_172232	-77	0.16
PF00005	ABC_tran	ABCA12	NM_173076	-62.89	0.18
PF00041	fn3	ITGB4	NM_000213	-151.66	0.09

PF00041	fn3	MYBPC3	NM_000256	-159.31	0.11
PF00041	fn3	TEK	NM_000459	-126.49	0.09
PF00041	fn3	NFASC	NM_001005388	-170.46	0.12
PF00041	fn3	MYBPHL	NM_001010985	-165.14	0.16
PF00041	fn3	NRCAM	NM_001037132	-172.56	0.09
PF00041	fn3	FNDC3A	NM_001079673	-167.66	0.1
PF00041	fn3	PTPRB	NM_001109754	-131.6	0.14
PF00041	fn3	SDK2	NM_001144952	-154.53	0.11
PF00041	fn3	PTPRQ	NM_001145026	-145.19	0.15
PF00041	fn3	IGFN1	NM_001164586	-142.77	0.14
PF00041	fn3	TTN	NM_001267550	-190.48	0.1
PF00041	fn3	DSCAM	NM_001389	-147.99	0.13
PF00041	fn3	NEO1	NM_002499	-145.11	0.08
PF00041	fn3	PTPRD	NM_002839	-174.45	0.17
PF00041	fn3	PTPRF	NM_002840	-173.09	0.13
PF00041	fn3	PTPRS	NM_002850	-172.94	0.05
PF00041	fn3	ROBO1	NM_002941	-160.15	0.07
PF00041	fn3	MYOM1	NM_003803	-165.32	0.18
PF00041	fn3	MYOM2	NM_003970	-162.48	0.11
PF00041	fn3	NCAM2	NM_004540	-71.64	0.2
PF00041	fn3	IGDCC3	NM_004884	-141.27	0.09
PF00041	fn3	CNTN2	NM_005076	-171.16	0.17
PF00041	fn3	DCC	NM_005215	-151.91	0.06
PF00041	fn3	TIE1	NM_005424	-142.77	0.18
PF00041	fn3	CHL1	NM_006614	-169.24	0.22
PF00041	fn3	CNTN5	NM_014361	-158.45	0.08
PF00041	fn3	EPHB2	NM_017449	-168.64	0.14
PF00041	fn3	IGDCC4	NM_020962	-159.35	0.15
PF00041	fn3	FNDC3B	NM_022763	-168.46	0.08
PF00041	fn3	FNDC1	NM_032532	-137.39	0.12
PF00041	fn3	CNTFR	NM_147164	-130.18	0.08
PF00041	fn3	FNDC5	NM_153756	-133.65	0.06
PF00041	fn3	PRTG	NM_173814	-140.57	0.08
PF00153	Mito_carr	SLC25A20	NM_000387	-208.62	0.07
PF00153	Mito_carr	SLC25A30	NM_001010875	-200.5	0.03
PF00153	Mito_carr	SLC25A29	NM_001039355	-197.36	0.02
PF00153	Mito_carr	SLC25A36	NM_001104647	-195.79	0.05
PF00153	Mito_carr	SLC25A19	NM_001126121	-193.8	0.15
PF00153	Mito_carr	SLC25A4	NM_001151	-191.48	0.07
PF00153	Mito_carr	SLC25A5	NM_001152	-190.04	0.04
PF00153	Mito_carr	SLC25A22	NM_001191061	-188.64	0.1
PF00153	Mito_carr	SLC25A14	NM_001282195	-186.79	0.06
PF00153	Mito_carr	SLC25A6	NM_001636	-128.79	0.06

PF00153	Mito_carr	UCP3	NM_003356	-117.26	0.07
PF00153	Mito_carr	SLC25A11	NM_003562	-115.84	0.08
PF00153	Mito_carr	SLC25A27	NM_004277	-113.07	0.14
PF00153	Mito_carr	SLC25A1	NM_005984	-112.5	0.1
PF00153	Mito_carr	SLC25A10	NM_012140	-109.96	0.08
PF00153	Mito_carr	SLC25A24	NM_013386	-80.41	0.25
PF00153	Mito_carr	SLC25A15	NM_014252	-74.29	0.14
PF00153	Mito_carr	SLC25A37	NM_016612	-73.61	0.21
PF00153	Mito_carr	SLC25A38	NM_017875	-64.12	0.17
PF00153	Mito_carr	UCP1	NM_021833	-64.12	0.22
PF00153	Mito_carr	SLC25A23	NM_024103	-58.03	0.12
PF00153	Mito_carr	SLC25A21	NM_030631	-57.96	0.16
PF00153	Mito_carr	SLC25A32	NM_030780	-57.76	0.08
PF00153	Mito_carr	SLC25A28	NM_031212	-54.49	0.3
PF00153	Mito_carr	SLC25A31	NM_031291	-52.02	0.3
PF00153	Mito_carr	SLC25A18	NM_031481	-47.66	0.19
PF00153	Mito_carr	SLC25A33	NM_032315	-46.07	0.41
PF00153	Mito_carr	SLC25A16	NM_152707	-44.21	0.28
PF00153	Mito_carr	SLC25A42	NM_178526	-40.58	0
PF00271	Helicase_C	BLM	NM_000057	-299.1	0.04
PF00271	Helicase_C	ERCC6	NM_000124	-277.54	0.06
PF00271	Helicase_C	WRN	NM_000553	-268.76	0.05
PF00271	Helicase_C	CHD3	NM_001005273	-299.63	0.06
PF00271	Helicase_C	DDX59	NM_001031725	-228.5	0.07
PF00271	Helicase_C	SHPRH	NM_001042683	-229.44	0.06
PF00271	Helicase_C	CHD8	NM_001170629	-301.35	0.03
PF00271	Helicase_C	CHD1	NM_001270	-300.98	0.03
PF00271	Helicase_C	CHD2	NM_001271	-299.61	0.04
PF00271	Helicase_C	CHD4	NM_001273	-300.57	0.03
PF00271	Helicase_C	SMARCA1	NM_001282874	-305.28	0.05
PF00271	Helicase_C	DDX46	NM_001300860	-253.67	0.04
PF00271	Helicase_C	CHD9	NM_001308319	-296.04	0.05
PF00271	Helicase_C	DDX3X	NM_001356	-300.07	0.02
PF00271	Helicase_C	EIF4A1	NM_001416	-298.87	0.06
PF00271	Helicase_C	EIF4A2	NM_001967	-303.25	0.03
PF00271	Helicase_C	RECQL	NM_002907	-294.28	0.05
PF00271	Helicase_C	SMARCA2	NM_003070	-293.15	0.03
PF00271	Helicase_C	HLTF	NM_003071	-252.33	0.06
PF00271	Helicase_C	SMARCA4	NM_003072	-297.34	0.02
PF00271	Helicase_C	SUPV3L1	NM_003171	-235.03	0.04
PF00271	Helicase_C	RAD54L	NM_003579	-276.68	0.04
PF00271	Helicase_C	TTF2	NM_003594	-247.12	0.07
PF00271	Helicase_C	SMARCA5	NM_003601	-236.77	0.05

PF00271	Helicase_C	BTAF1	NM_003972	-270.76	0.02
PF00271	Helicase_C	RECQL5	NM_004259	-280.16	0.06
PF00271	Helicase_C	RECQL4	NM_004260	-106.06	0.14
PF00271	Helicase_C	CHD1L	NM_004284	-268.4	0.05
PF00271	Helicase_C	DDX5	NM_004396	-88.56	0.06
PF00271	Helicase_C	DDX6	NM_004397	-66.89	0.07
PF00271	Helicase_C	DDX10	NM_004398	-223.36	0.08
PF00271	Helicase_C	DDX39B	NM_004640	-78.61	0.07
PF00271	Helicase_C	DDX3Y	NM_004660	-299.93	0.04
PF00271	Helicase_C	DDX21	NM_004728	-279.1	0.1
PF00271	Helicase_C	DDX23	NM_004818	-263.73	0.05
PF00271	Helicase_C	DDX1	NM_004939	-247.1	0.04
PF00271	Helicase_C	DDX39A	NM_005804	-78.07	0.06
PF00271	Helicase_C	DDX17	NM_006386	-91.14	0.04
PF00271	Helicase_C	SRCAP	NM_006662	-278.39	0.35
PF00271	Helicase_C	DDX18	NM_006773	-81.54	0.07
PF00271	Helicase_C	ASCC3	NM_006828	-262.39	0.1
PF00271	Helicase_C	DDX52	NM_007010	-99.4	0.14
PF00271	Helicase_C	DDX20	NM_007204	-39.83	0.37
PF00271	Helicase_C	DDX19B	NM_007242	-266.24	0.05
PF00271	Helicase_C	RAD54B	NM_012415	-276.32	0.07
PF00271	Helicase_C	DDX25	NM_013264	-261.78	0.16
PF00271	Helicase_C	SMARCAL1	NM_014140	-249.88	0.09
PF00271	Helicase_C	DDX58	NM_014314	-209.3	0.11
PF00271	Helicase_C	DHX34	NM_014681	-287.87	0.06
PF00271	Helicase_C	EIF4A3	NM_014740	-280.11	0.02
PF00271	Helicase_C	RAD54L2	NM_015106	-255.93	0.02
PF00271	Helicase_C	EP400	NM_015409	-53.93	0.27
PF00271	Helicase_C	CHD5	NM_015557	-297.71	0.06
PF00271	Helicase_C	DDX41	NM_016222	-210.75	0.04
PF00271	Helicase_C	DDX47	NM_016355	-279.38	0.05
PF00271	Helicase_C	INO80	NM_017553	-279.83	0.01
PF00271	Helicase_C	ERCC6L	NM_017669	-254.26	0.08
PF00271	Helicase_C	CHD7	NM_017780	-302.78	0.12
PF00271	Helicase_C	DDX27	NM_017895	-263.97	0.11
PF00271	Helicase_C	HELLS	NM_018063	-289.77	0.05
PF00271	Helicase_C	DDX19A	NM_018332	-266.36	0.04
PF00271	Helicase_C	DDX28	NM_018380	-212.42	0.04
PF00271	Helicase_C	DDX43	NM_018665	-244.15	0.06
PF00271	Helicase_C	DDX49	NM_019070	-244.57	0.05
PF00271	Helicase_C	SMARCAD1	NM_020159	-254.15	0.05
PF00271	Helicase_C	ERCC6L2	NM_020207	-244.94	0.06
PF00271	Helicase_C	DDX24	NM_020414	-72.64	0.12

PF00271	Helicase_C	DDX55	NM_020936	-213.06	0.07
PF00271	Helicase_C	FANCM	NM_020937	-269.92	0.06
PF00271	Helicase_C	IFIH1	NM_022168	-232.61	0.05
PF00271	Helicase_C	DDX50	NM_024045	-271.78	0.04
PF00271	Helicase_C	DDX54	NM_024072	-248.89	0.07
PF00271	Helicase_C	DDX4	NM_024415	-274.02	0.07
PF00271	Helicase_C	ZRANB3	NM_032143	-254.67	0.09
PF00271	Helicase_C	CHD6	NM_032221	-299.01	0.03
PF00271	Helicase_C	DDX51	NM_175066	-228.6	0.07
PF00271	Helicase_C	DICER1	NM_177438	-198.51	0.02
PF00271	Helicase_C	DDX53	NM_182699	-178.59	0.26
PF00271	Helicase_C	DDX42	NM_203499	-262.43	0.02
